# People with genetic kidney diseases on kidney replacement therapy have different clinical outcomes compared to people with other kidney diseases

**DOI:** 10.1038/s41598-024-57273-x

**Published:** 2024-03-21

**Authors:** Helen Y. Han, Venkat Vangaveti, Matthew Jose, Monica Suet Ying Ng, Andrew John Mallett

**Affiliations:** 1 School of Medicine, The University of Tasmania, Hobart, TAS Australia; 2 Kidney Health Service, Royal Brisbane and Women’s Hospital, Butterfield Street, Herston, QLD 4029 Australia; 3 College of Medicine and Dentistry, James Cook University, Townsville, QLD Australia; 4 Townsville Institute of Health Research and Innovation, Townsville University Hospital, Douglas, QLD Australia; 5 Hobart Clinical School, University of Tasmania, Hobart, TAS Australia; 6 Conjoint Internal Medicine Laboratory, Chemical Pathology, Health Support Queensland Pathology Queensland, Brisbane, QLD Australia; 7 Faculty of Medicine, The University of Queensland, Brisbane, QLD Australia; 8 Institute for Molecular Biosciences, University of Queensland, Brisbane, QLD Australia

**Keywords:** Registry, Genetic kidney disease, Mortality, Kidney failure, Kidney transplant, Dialysis, Renal replacement therapy, Kidney diseases, Chronic kidney disease

## Abstract

Despite increasing awareness of genetic kidney disease prevalence, there is limited population-level information about long term outcomes of people with genetic kidney disease receiving kidney replacement therapy. This analysis included people who commenced kidney replacement therapy between 1989 and 2020 as recorded in the Australian and New Zealand Dialysis and Transplant registry. Genetic kidney diseases were subclassified as majority and minority monogenic. Non-genetic kidney diseases were included as the comparator group. Primary outcome measures were 10-year mortality and 10-year graft failure. Cox proportional hazard regression were used to calculate unadjusted and adjusted hazard ratios (AHRs) for primary outcomes. There were 59,231 people in the dialysis subgroup and 21,860 people in the transplant subgroup. People on dialysis with genetic kidney diseases had reduced 10-year mortality risk (majority monogenic AHR: 0.70, 95% CI 0.66–0.76; minority monogenic AHR 0.86, 95% CI 0.80–0.92). This reduced 10-year mortality risk continued after kidney transplantation (majority monogenic AHR: 0.82, 95% CI 0.71–0.93; minority monogenic AHR 0.80, 95% CI 0.68–0.95). Majority monogenic genetic kidney diseases were associated with reduced 10-year graft failure compared to minority monogenic genetic kidney diseases and other kidney diseases (majority monogenic AHR 0.69, 95% CI 0.59–0.79). This binational registry analysis identified that people with genetic kidney disease have different mortality and graft failure risks compared to people with other kidney diseases.

## Introduction

Genetic kidney diseases (GKDs) are among the leading causes of early-onset chronic kidney disease and responsible for more than 10–15% cases of kidney failure requiring kidney replacement therapy (KRT)^1^. GKDs also contribute significantly to kidney disease of unknown aetiology cohorts. In an Irish non-polycystic chronic kidney disease population, 26.9% patients reported a positive family history and the commonest aetiology in the positive family history cohort was kidney disease due to unknown aetiology^2^. Exome sequencing identified diagnostic variants amongst 17.1% of those with kidney disease of unknown origin across 3037 patients with chronic kidney disease^3^. Despite increasing awareness of GKD burden, there is limited population-level information about long term outcomes of people with GKD receiving KRT^3,4^.

The Australia and New Zealand Dialysis and Transplant (ANZDATA), United States Renal Data System (USRDS) and European Renal Association – European Dialysis and Transplantation Association (ERA-EDTA) Registry reports present GKDs as “Polycystic Disease” and “Other”^5–8^. The former category excludes non-cystic GKD and while the latter category conflates non-cystic GKDs with other non-genetic causes of kidney failure. Available longitudinal studies of GKDs are limited to single centres and/or single diseases^9,10^. These provide limited information about GKD outcomes overall and are not able to compare GKD outcomes to those of the non-GKD population. Yet, it is clear that people with GKD on KRT are demographically different (e.g. younger at time of KRT initiation, less comorbidities) with multi-system diseases (e.g. autosomal dominant polycystic kidney disease, Fabry disease)^10,11^.

There is an unmet need to profile long term KRT outcomes in the GKD population to inform patient prognostication, counselling and resource allocation. This study aimed to characterise clinical outcomes (10-year mortality, 10-year graft failure) in people with GKD receiving KRT and compare them to those of people with other kidney diseases.

## Results

### Patient demographics

**Figure 1:**
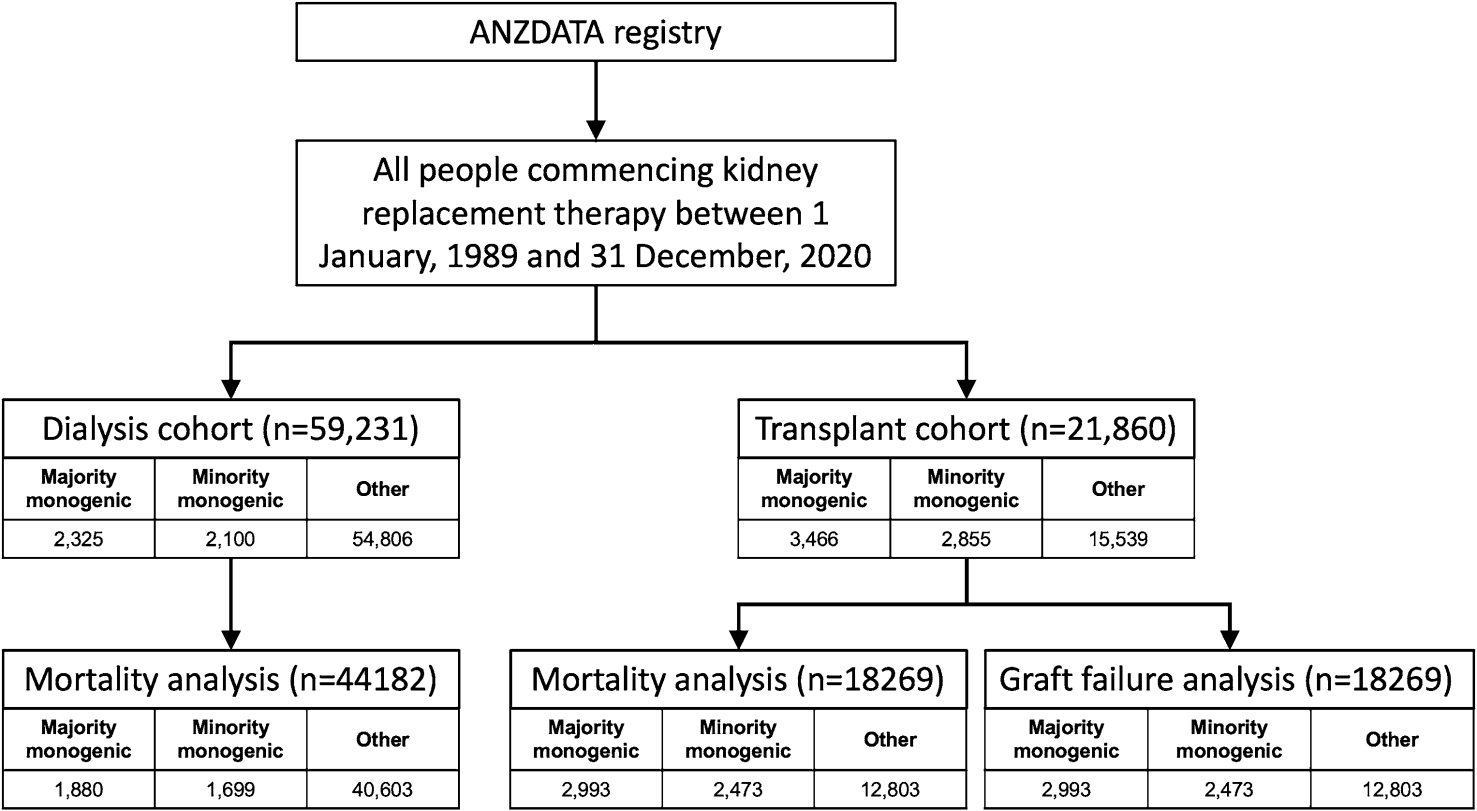
Flowchart of people included in study.

The study cohort included 59,231 patients in the dialysis subgroup and 21,860 in the transplant subgroup (Fig. 1). The prevalence of GKDs was 7.4% of the dialysis population with 2325 (3.9%) having majority monogenic GKDs and 2100 (3.5%) having minority monogenic GKDs and 54,806 (92.5%) having other kidney diseases. In the transplanted population, the prevalence of GKDs was 29% with 3466 (15.9%) having majority monogenic GKDs, 2855 (13.1%) having minority monogenic GKDs and 15,539 (71.1%) having other kidney diseases. Mean follow-up time was 9.5 years (SD: 7.23) for majority monogenic GKDs, 10.36 years (SD: 8.39) for minority monogenic GKDs and 5.63 years (SD: 5.90) for other kidney diseases. The constituents of each kidney disease group – majority monogenic, minority monogenic and other kidney diseases are presented in Supplementary Table S1.

In the dialysis cohort, people with GKDs were more likely to be female, younger, never smokers and white with body bass indices between 18.5 and 24.9 and peritoneal dialysis as first KRT modality compared to other kidney diseases (Table 1). People with GKD on dialysis were less likely to have peripheral vascular disease, chronic lung disease, coronary artery disease, cerebrovascular disease. People with GKD had reduced 1-year, 3-year and 5-year mortality rates compared to people with other kidney diseases (Table 1). The distribution of cause of mortality was similar between GKD and other kidney diseases.

**Table 1:** Socio-demographic characteristics of the dialysis cohort.

Characteristics	Majority monogenic, N = 2325	Minority monogenic, N = 2100	Other, N = 54,806	P-value
Age (years)				< 0.001***
< 20	17 (0.7)	75 (3.6)	172 (0.3)	
20–39	119 (5.1)	350 (16.7)	2953 (5.4)	
40–59	743 (32.0)	664 (31.6)	15,243 (27.8)	
60–79	1335 (57.4)	927 (44.1)	31,664 (57.7)	
80 +	111 (4.8)	84 (4.0)	4794 (8.7)	
Gender				< 0.001***
Female	1051 (45.2)	1041 (49.6)	21,822 (39.8)	
Male	1274 (54.8)	1059 (50.4)	32,984 (60.2)	
Smoking status				< 0.001***
Current	286 (12.3)	391 (18.6)	7247 (13.2)	
Former	851 (36.6)	684 (32.6)	22,605 (41.2)	
Never	1118 (48.1)	964 (45.9)	23,321 (42.6)	
Missing	70 (3.0)	61 (2.9)	1633 (3.0)	
BMI (kg/m^2^)				< 0.001***
< 18.5	77 (3.3)	135 (6.4)	1625 (3.0)	
18.5–24.9	891 (38.3)	677 (32.2)	16,162 (29.5)	
25–29.9	695 (29.9)	541 (25.8)	16,030 (29.5)	
> 30	520 (22.4)	592 (28.2)	17,116 (31.2)	
Missing	142 (6.1)	155 (7.4)	3873 (7.1)	
Ethnicity				< 0.001***
White	2020 (86.9)	1524 (72.6)	35,862 (65.4)	
ATSI	30 (1.3)	137 (6.5)	5536 (10.1)	
Maori	50 (2.2)	158 (7.5)	4143 (7.6)	
Asian	121 (5.2)	129 (6.1)	4424 (8.1)	
Other	96 (4.1)	144 (6.9)	4473 (8.2)	
Missing	8 (0.3)	8 (0.4)	368 (0.7)	
Comorbidities at KRT entry				
Peripheral vascular disease	170 (7.3)	167 (8.0)	12,051 (22.0)	< 0.001***
Chronic lung disease	220 (9.5)	292 (13.9)	8172 (14.9)	< 0.001***
Coronary artery disease	570 (24.5)	494 (23.5)	20,739 (37.8)	< 0.001***
Cerebrovascular disease	243 (10.5)	179 (8.5)	7014 (12.8)	< 0.001***
Diabetes mellitus	269 (11.6)	409 (19.5)	30,014 (54.8)	< 0.001***
First dialysis modality				< 0.001***
Haemodialysis	1557 (67.0)	1445 (68.8)	40,011 (73.0)	
Peritoneal dialysis	768 (33.0)	655 (31.2)	14,795 (27.0)	
Dialysis vintage				< 0.001***
1989–1998	486 (20.9)	336 (16.0)	8536 (15.6)	
1999–2008	645 (27.7)	585 (27.9)	17,163(31.3)	
2009–2018	852 (36.6)	823 (39.2)	22,597 (41.2)	
2018–2021	342 (14.7)	356 (17.0)	6510 (11.9)	
Mortality rate				
1 year mortality	331 (14.2)	370 (17.6)	14,332 (26.2)	< 0.001***
3 year mortality	672 (28.9)	662 (31.5)	25,069 (45.7)	< 0.001***
5 year mortality	945 (40.6)	892 (42.5)	31,681 (57.8)	< 0.001***
Cause of mortality^a^				< 0.001***
Cardiovascular	540 (36.9)	449 (35.8)	14,175 (35.6)	
Infection	158 (10.8)	147 (11.7)	4421 (11.1)	
Withdrawal	406 (27.8)	326 (26.0)	10,487 (26.3)	
Cancer	72 (4.9)	68 (5.4)	1885 (4.7)	
Other	284 (19.4)	257 (20.5)	8589 (21.6)	
Missing (did not die)	3 (0.2)	6 (0.5)	253 (0.6)	

*ATSI* aboriginal and torres strait islander, *BMI* body mass index, *kg* kilogram, *KRT* kidney replacement therapy, *m* metre.

Significance level: *** < 0.001.

^a^Percentage expressed as proportion of people who died during follow-up.

In the transplant cohort, people with GKD were more likely to be female, never smokers, white with peritoneal dialysis as first KRT modality (Table 2). People with GKD and kidney transplant were less likely to have peripheral vascular disease, coronary artery disease, chronic lung disease and diabetes mellitus. Mean dialysis vintage, HLA mismatch and cold ischaemia time were similar between people with GKD and other kidney diseases. 1 yearmortality and graft failure rates were similar between people with GKD and other kidney diseases. People with GKD have similar rates of graft failure secondary to rejection, chronic allograft nephropathy, vascular issues, technical issues, non-compliance and other. There was reduced graft failure secondary to glomerulonephritis in kidney transplant recipients with majority monogenic GKD.

**Table 2:** Socio-demographic characteristics of the transplant cohort.

Characteristics	Majority monogenic, N = 3466	Minority monogenic, N = 2855	Other, N = 15,539	P-value
Recipient age (years)				< 0.001***
< 20	208 (6.0)	560 (19.6)	886 (5.7)	
20–39	499 (14.4)	1153 (40.4)	4565 (29.4)	
40–59	2190 (63.2)	950 (33.3)	7477 (48.1)	
60–79	569 (16.4)	192 (6.7)	2610 (16.8)	
80 +	0 (0.0)	0 (0.0)	1 (0.0)	
Recipient gender				< 0.001***
Female	1510 (43.6)	1307 (45.8)	5396 (34.7)	
Male	1956 (56.4)	1548 (54.2)	10,143 (65.3)	
Recipient smoking status				< 0.001***
Never	2062 (59.5)	1806 (63.3)	8539 (55.0)	
Former	1050 (30.3)	667 (23.4)	4902 (31.5)	
Current	254 (7.3)	303 (10.6)	1497 (9.6)	
Missing	100 (2.9)	79 (2.8)	601 (3.9)	
Recipient BMI (kg/m^2^)				< 0.001***
< 18.5	187 (5.4)	364 (12.7)	824 (5.3)	
18.5–24.9	1355 (39.1)	1173 (41.1)	5736 (36.9)	
25–29.9	1121 (32.3)	706 (24.7)	4627 (29.8)	
> 30	600 (17.3)	425 (14.9)	3254 (20.9)	
Missing	203 (5.9)	187 (6.5)	1098 (7.1)	
Recipient ethnicity				< 0.001***
White	3002 (86.6)	2254 (78.9)	10,779 (69.4)	
ATSI	20 (0.6)	68 (2.4)	646 (4.2)	
Maori	30 (0.9)	71 (2.5)	506 (3.3)	
Asian	158 (4.6)	177 (6.2)	1987 (12.8)	
Other	246 (7.1)	272 (9.5)	1474 (9.5)	
Missing	10 (0.3)	13 (0.5)	147(0.9)	
Recipient comorbidities				
Peripheral vascular disease	46 (1.3)	29 (1.0)	822 (5.3)	< 0.001***
Coronary artery disease	223 (6.4)	106 (3.7)	1487 (9.6)	< 0.001***
Chronic lung disease	88 (2.5)	86 (3.0)	647 (4.2)	< 0.001***
Cerebrovascular disease	119 (3.4)	31 (1.1)	469 (3.0)	< 0.001***
Diabetes mellitus	111 (3.2)	107 (3.7)	4074 (26.2)	< 0.001***
First KRT modality				< 0.001***
Haemodialysis	2025 (58.4)	1449 (50.8)	9338 (60.1)	
Peritoneal dialysis	918 (26.5)	916 (32.1)	4658 (30.0)	
Pre-emptive transplant	523 (15.1)	490 (17.2)	1543 (9.9)	
Dialysis vintage (yr, mean, SD)	1.84 (2.44)	2.07 (2.91)	2.23 (2.68)	< 0.001***
HLA mismatch (mean, SD)	3.29 (1.63)	3.09 (1.65)	3.33 (1.64)	< 0.001***
Cold ischemia time (hr, mean, SD)	9.36 (6.31)	8.73 (6.65)	9.86 (6.46)	< 0.001***
Donor age				< 0.001***
< 20	300 (8.7)	270 (9.5)	1603 (10.4)	
20–39	731 (21.1)	747 (26.2)	3989 (25.9)	
40–59	1750 (50.6)	1405 (49.3)	6849 (44.4)	
60 & above	678 (19.6)	427 (15.0)	2975 (19.3)	
Donor source				< 0.001***
Deceased	2261 (65.2)	1665 (58.3)	10,848 (69.8)	
Live donor	1205 (34.8)	1190 (41.7)	4961 (30.2)	
Transplant era				< 0.001***
1989–1998	540 (15.6)	607 (21.3)	2620 (16.9)	
1999–2008	1007 (29.1)	850 (29.8)	4462 (28.7)	
2009–2018	1607 (46.4)	1147 (40.2)	6876 (44.2)	
2018–2021	312 (9.0)	251 (8.8)	1581 (10.2)	
Mortality rate				
1 year mortality	39 (1.1)	17 (0.6)	176 (1.1)	0.47
3 year mortality	88 (2.5)	47 (1.6)	539 (3.5)	0.01**
5 year mortality	144 (4.2)	81 (2.8)	961 (6.2)	< 0.001***
Cause of mortality^a^				< 0.001***
Cardiovascular	212 (24.3)	143 (26.5)	1382(31.5)	
Infection	137 (15.7)	91 (16.9)	723 (16.5)	
Withdrawal	79 (9.0)	60 (11.1)	416 (9.5)	
Cancer	242 (27.7)	101 (18.7)	840 (19.2)	
Other	191 (21.9)	141 (26.2)	993 (22.7)	
Missing	12 (1.4)	3 (0.6)	30(0.7)	
Graft failure rate				
1 year graft failure	109 (20.1)	161 (19.5)	728 (21.8)	0.30
3 year graft failure	162 (29.9)	250 (30.3)	1129 (66.2)	0.06
5 year graft failure	217 (40.0)	343 (41.6)	1527 (45.7)	0.01**
Cause of graft failure^b^				< 0.001***
Rejection (acute + hyperacute)	46 (8.9)	56 (7.0)	243 (7.5)	
Chronic allograft nephropathy	297 (57.3)	464 (57.8)	1743 (53.5)	
Vascular	42 (8.1)	60 (7.5)	230 (7.1)	
Technical	14 (2.7)	18 (2.2)	73 (2.2)	
Glomerulonephritis	14 (2.7)	50 (6.2)	276 (8.5)	
Non-compliance	16 (3.1)	43 (5.4)	155 (4.8)	
Other	89 (17.2)	112 (13.9)	536 (16.5)	
Disease in graft kidney				< 0.001***
BK virus nephropathy	74 (2.1)	55 (1.9)	390 (2.5)	
De novo glomerulonephritis	33 (1.0)	44 (1.5)	119 (0.8)	
Glomerulonephritis in graft	8 (0.2)	88 (3.1)	508 (3.3)	
Disease recurrence	4 (0.1)	5 (0.2)	85 (0.5)	

*ATSI* aboriginal and torres strait islander, *BMI* body mass index, *HLA* human leukocyte antigen, *hr* hour, *kg* kilogram, *KRT* kidney replacement therapy, *m* metre, *SD* standard deviation, *yr* year.

Significance level: ** < 0.01, *** < 0.001.

^a^Percentage expressed as proportion of people who died during follow-up.

^b^Percentage expressed as proportion of people who had graft failure during follow-up.

### Mortality

**Figure 2:**
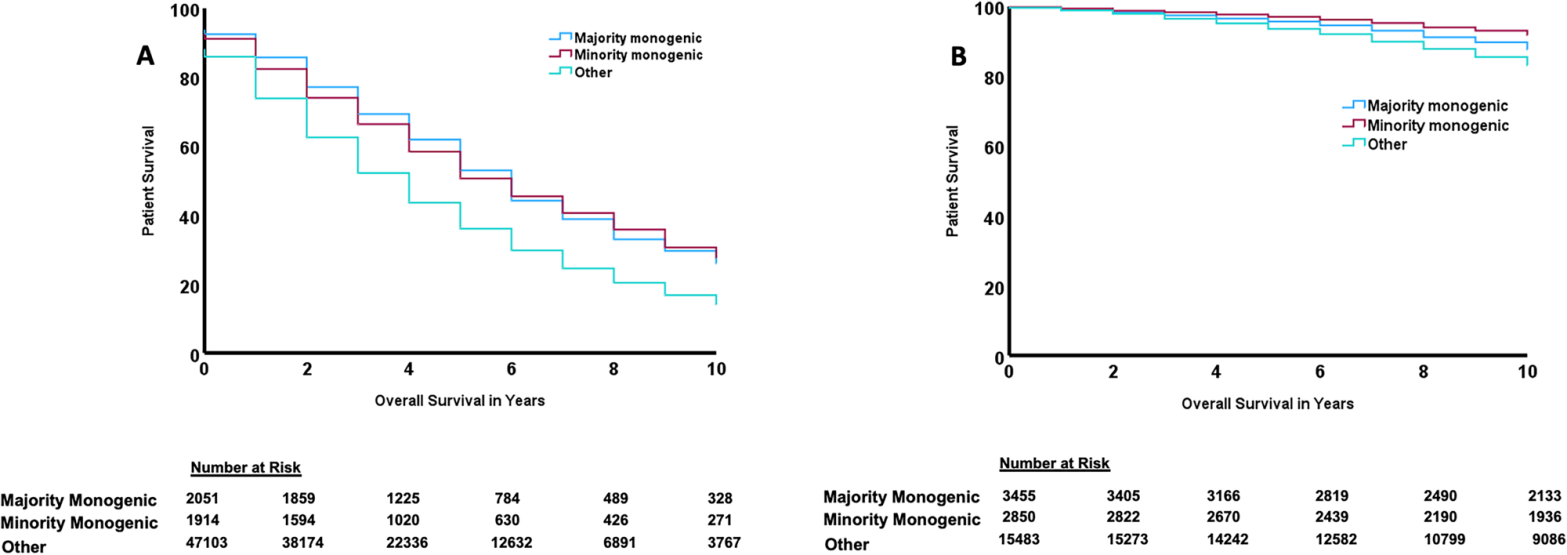
Unadjusted Kaplan Meier curves for patient survival after starting kidney replacement therapy – (**A**) dialysis, (**B**) kidney transplant.

On Kaplan Meier analyses, majority monogenic and minority monogenic GKD had reduced 10-year mortality risk compared to other kidney disease in people who remained on dialysis (Fig. 2A). This finding was confirmed on univariable and multivariable Cox proportional hazard models (majority monogenic AHR: 0.70, 95% CI 0.66–0.76; minority monogenic AHR 0.86, 95% CI 0.80–0.92; Table 3, Supplementary Table S2).

**Table 3:** Unadjusted + adjusted hazard ratios + 95% CI for association between genetic kidney disease and 10-year mortality in dialysis cohort.

Effect	Unadjusted		Adjusted	
	HR	95% CI	AHR	95% CI
Disease status				
Majority monogenic	0.67***	0.64–0.72	0.70***	0.66–0.76
Minority monogenic	0.67***	0.63–0.72	0.86***	0.80–0.92
Other kidney diseases	Ref		Ref	

*AHR* adjusted hazard ratio, *HR* hazard ratio.

Significance level: *** < 0.001.

On Kaplan Meier analyses, kidney transplant recipients with minority monogenic and majority monogenic GKD had reduced 10-year mortality risk compared to other kidney disease (Fig. 2B). Minority monogenic and majority monogenic GKDs were associated with reduced 10-year mortality risk in kidney transplant recipients on multivariable analyses (majority monogenic AHR 0.82, 95% CI 0.71–0.93; minority monogenic AHR 0.80, 95% 0.68–0.95; Table 4, Supplementary Table S3).Graft failure-censored mortality was reduced in transplant recipients with majority monogenic GKD compared to other kidney diseases (AHR 0.84, 95% CI 0.76–0.93, Supplementary Table S4).

**Table 4:** Unadjusted + adjusted hazard ratios + 95% CI for association between genetic kidney disease and 10 year mortality in transplant cohort.

Effect	Unadjusted		Adjusted	
	HR	95% CI	AHR	95% CI
Disease status				
Majority monogenic	0.70***	0.62–0.79	0.82**	0.71–0.93
Minority monogenic	0.46***	0.40–0.53	0.80**	0.68–0.95
Other kidney diseases	Ref		Ref	

*AHR* adjusted hazard ratio, *HR* hazard ratio; *NA* not applicable.

Significance level: ** < 0.01, *** < 0.001.

### Graft failure

**Figure 3:**
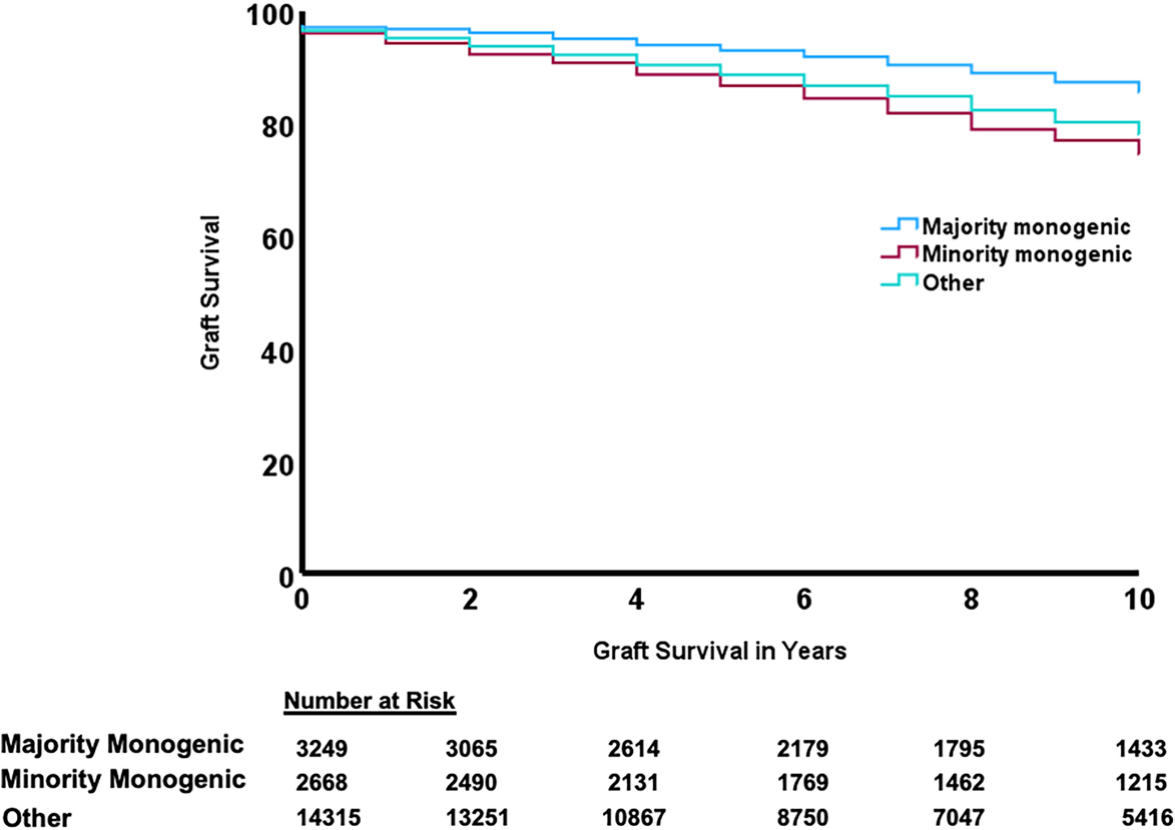
Unadjusted Kaplan Meier curves for graft survival after kidney transplant.

On Kaplan Meier analyses, majority monogenic and minority monogenic GKDs had reduced 10-year graft failure risk compared to and other kidney diseases (Fig. 3). Only majority monogenic GKD correlated with reduced 10-year graft failure compared to other kidney diseases on multivariable analysis (AHR 0.69, 95% CI 0.59–0.79; Table 5, Supplementary Table S5). This was confirmed on competing risk analyses where mortality-censored graft failure was reduced in people with majority monogenic GKDs compared to other kidney diseases (AHR 0.77, 95% CI 0.68–0.87, Supplementary Table S4).

**Table 5:** Unadjusted + adjusted hazard ratios + 95% CI for association between genetic kidney disease and 10-year graft failure.

Effect	Unadjusted		Adjusted	
	HR	95% CI	AHR	95% CI
Disease status				
Majority monogenic	0.57***	0.50–0.65	0.69***	0.59–0.79
Minority monogenic	1.20***	1.08–1.33	1.10	0.98–1.24
Other kidney diseases	Ref		Ref	

*AHR* adjusted hazard ratio, *HR* hazard ratio, *NA* not applicable.

Significance level: *** < 0.001.

## Discussion

This binational registry study longitudinally followed outcomes of people with GKD from KRT initiation to graft failure and/or death. Previous studies of people with GKD and kidney failure have focused on individual disease outcomes^12,13^ and utility of genetic testing in cases of suspected GKD or unknown cause of kidney failure^1,3^. Our study provided a population-level overview of all GKDs and compared clinical outcomes to those of people with other kidney diseases – providing key information to guide patient counselling, health policy and resource allocation.

GKD-specific information is necessary because GKD demographics are substantially different to those of people with other kidney diseases. Similar to other studies^9,10,12^, people with GKD receiving KRT tended to be younger with less comorbidities, no smoking history and normal body habitus (Tables 1 and 2). Our study further subdivided GKDs into majority and minority monogenic based on the percentage of cases with identifiable monogenic bases – providing further clarification based on concentration of GKDs within each group.

In the dialysis cohort, there was reduced 10-year mortality risk in people with majority and minority monogenic GKDs after controlling for age, gender, ethnicity, smoking status, BMI, comorbidities, dialysis mortality and dialysis vintage. There was a dose-dependent effect with majority monogenic GKDs having the lowest AHR of 0.70 and 95% CI of 0.66–0.76. This reduced mortality risk was also observed on subgroup analyses confirming that this effect was consistent across multiple GKDs and not just polycystic kidney disease and reflux nephropathy (Supplementary Table S6). This reduced mortality risk was consistent with previously analyses of United States Renal Data System^13^ and Taiwan’s National Health Insurance data^14^. Further research is required to identify the unmeasured socioeconomic, nutritional and environmental factors contributing to mortality differences in people with GKD compared to other kidney diseases.

Kidney transplant recipients with majority monogenic and minority monogenic GKD had reduced 10-year mortality risk compared to people with other kidney diseases. On subgroup analyses, the reduced 10-year mortality risk was only observed in people with polycystic kidney disease and reflux nephropathy (Supplementary Table S7). The reduced mortality risk seen in people in polycystic kidney disease align with analyses of kidney transplant recipients at Mayo Clinic where median post-transplant survival was 18.7 years for people with polycystic kidney disease and 13.8 years for people with non-diabetic other kidney disease^15^. Retrospective analysis of 745 kidney transplants completed in Turkey demonstrated people with reflux nephropathy had similar mortality risk compared to those with chronic glomerular disease or unknown aetiologies^16^. Notably, our analysis compared people with reflux nephropathy to people with other non-genetic kidney diseases including diabetic kidney disease which may explain our different results as people with diabetes are known to have reduced overall survival compared to non-diabetic people^17^. Further research is required to confirm disease-associated differences in social determinants of health amongst people with kidney failure.

There was reduced 10-year graft failure in people with majority monogenic GKD compared to minority monogenic GKD and other kidney diseases on multivariable analysis. On subgroup analysis, this result was primarily attributed to reduced graft failure in people with polycystic kidney disease (Supplementary Table S8) This is consistent with studies demonstrating that people with kidney failure secondary to autosomal dominant polycystic kidney disease^13,18^ have reduced graft failure compared to people with other kidney diseases. These differences can be attributed to negligible risks of disease recurrence. Other proposed theories include reduced alloimmunisation against HLA antigens, potentially in the context of reduced blood transfusions and pregnancies^18^. Further studies are required to test this hypothesis.

The key limitation of this study include misclassification of GKDs, heterogenous GKD groups and effect of unmeasured confounders. Primary kidney diseases are primarily classified based on histological or clinical disease definitions in ANZDATA and we subclassified these into GKD subgroups based on likelihood of monogenic disease basis. As a result, polygenic GKDs were not assessed in this study. Some cases of kidney disease, particularly in the minority monogenic group, may not have a monogenic disease basis and are not true instances of GKD. Furthermore, classification of GKDs into majority and minority monogenic were based on current knowledge of GKDs. New genotype–phenotype associations are discovered frequently, potentially affecting the classification of kidney diseases. Notably and as an example, 969 new “green” genes were added to version 1 + panels used in the 100,000 Genomes Project with an average of 5.6 additional genes per panel four years after initial panel design^19^ Subsequent studies could investigate longitudinal outcomes of people with kidney failure secondary to sequencing-confirmed GKDs to confirm the findings of this study. Such a study may be limited by differential access to genetic testing across jurisdictions and limited genetic testing in historical populations.

The study sought to provide an overview of KRT outcomes in people with GKD. Thus, different GKDs were grouped into majority monogenic and minority monogenic GKDs. This approach masks the differential associations of different kidney diseases on KRT outcomes. While polycystic kidney disease has been associated with reduced mortality in dialysis and transplant populations; Fabry disease has been associated with increased mortality in dialysis and transplant populations^10^. Furthermore, the larger constituents of majority and minority monogenic GKDs such as polycystic kidney disease and reflux nephropathy may skew the results of the analyses (Supplementary Table S1). This was demonstrated for the graft failure analyses where the results of the polycystic kidney disease and reflux nephropathy aligned with those of the majority monogenic and minority monogenic GKD results in the main analysis (Supplementary Table S8). Notably, subgroup analyses confirmed that reduced mortality risk was observed for people on dialysis was consistent across polycystic kidney disease, other majority monogenic GKDs, reflux nephropathy and other minority monogenic GKDs (Supplementary Table S6). On subgroup analyses, polycystic kidney disease and reflux nephropathy correlated with reduced mortality risk in the transplant cohort (Supplementary Table S7).

This study was a registry analysis with shortcomings related to unmeasured confounders such as socioeconomic status, nutrition and environmental exposure. Future studies may address this issue by using database linkage methodologies. Lastly, our findings are primarily applicable to the Australian and New Zealand population and health infrastructure. Further studies using data from USRDS and ERA-EDTA registries are required to verify and replicate these results in other jurisdictions.

This comprehensive binational registry analysis of people with GKD receiving KRT identified that people with GKD have different 10-year mortality and 10-year graft failure risks compared to people with other kidney diseases. Those with GKD receiving dialysis had superior mortality risk compared to people with other kidney diseases. The reduced mortality risk was maintained after kidney transplant. People with majority monogenic GKDs had reduced graft failure compared to minority monogenic GKDs and other kidney diseases. Further studies are required to identify and characterise underlying socioeconomic and environmental factors which may contribute to these results.

## Methods

### Study design and setting

This retrospective observational study included people who commenced KRT between 1 January, 1989 and 31 December, 2020 as recorded in the ANZDATA registry. De-identified information on patient and graft donor variables were received from the ANZDATA registry which stores information on people on kidney replacement therapy in Australia and New Zealand (Tables 1 and 2). The study was approved by the Human Research Ethics Committee (HREC) of the University of Tasmania (20,409) and ANZDATA steering committee (42,745). All methods were performed in accordance with relevant guidelines and regulations. All individual participants provided informed consent on entry into ANZDATA registry for research approved by ANZDATA and local HREC.

### Study variables

Primary kidney disease codes in ANZDATA registry align with European Renal Association primary kidney disease codes. Primary kidney disease diagnoses are annotated by kidney specialist based on clinical assessment and are not always genetically- nor biopsy- proven. To enhance capture of GKDs, GKDs were subclassified as majority monogenic and minority monogenic based on percentage of cases within each disease classification with monogenic bases as previously described^20^ (Supplementary Table S1). Majority monogenic GKDs were defined by evidence from cohort/case studies that ≥ 50% cases have an identifiable monogenic bases. Minority monogenic were defined by evidence from cohort/case series studies that < 50% cases have an identifiable monogenic basis. Monogenic basis was defined as a likely pathogenic or pathogenic (ACMG variant classification) variant or variants with the appropriate zygosity in a gene with an established or justified gene-phenotype/kidney disease relationship. Phenocopy disorders were excluded from the assessment of monogenic basis for kidney disease. Evidence was drawn from Online Mendelian Inheritance in Man, PanelApp Australia and the ClinGen Clinical Domain Working Groups^21^. People with kidney diseases that had no monogenic basis were included as a comparator group. People were included in the dialysis cohort if they did not receive a kidney transplant by 31 December, 2020. People were included in the transplant cohort if they received a kidney transplant.

Primary outcome measures included 10-year mortality for the dialysis and transplant cohort; and 10-year graft failure for the transplant cohort. Graft failure occurred when the graft was no longer functioning (excluding death with functioning graft). Secondary outcome measures such as cause of death, cause of graft failure and disease in graft kidney were recorded for descriptive purposes. Patient age, gender, smoking status, body mass index (BMI), ethnicity, comorbidities (diabetes, chronic lung disease, coronary artery disease, peripheral vascular disease, cerebrovascular disease), first KRT modality, dialysis vintage were assessed for the dialysis cohort. Additional variables such as donor source, donor age, cold ischemia time, human leukocyte antigen (HLA) mismatch, dialysis vintage and transplant era were assessed for the transplant cohort.

### Statistical analysis

Categorical demographic variables were reported using counts and percentages (Tables 1 and 2). Continuous demographic variables were reported was means and standard deviations. Chi-squared test of independence were used for categorical variables and Analysis of Variance (ANOVA) were used to compared continuous variables.

Kaplan–Meier survival curves were used to assess time from dialysis initiation (dialysis cohort) or transplant date (transplant cohort) to death or graft failure. In the transplant cohort, Kaplan–Meier survival curves were used to visualize time from transplant date to graft failure. Cox proportional hazards regression were used to calculate unadjusted and adjusted hazard ratio (AHR) of mortality in the dialysis and transplant cohorts; and graft failure in the transplant cohort. Mortality and graft failure were assessed as time-to-event variables. In the dialysis cohort, disease type, age in decades, gender, ethnicity, smoking status, BMI, smoking status, comorbidities, first dialysis modality and dialysis vintage were included into the mortality model as covariates. In the transplant cohort, disease type, recipient age in decades, gender, ethnicity, smoking status, BMI, comorbidities, first dialysis modality, dialysis vintage in years; donor source and age in decades; cold ischemia time in hours, HLA mismatch, and transplant era were included into the mortality and graft failure models as covariates. Test for proportional hazard assumption were initially completed using log–log survival curves (Supplementary Fig. 1). Where log–log survival curves intersect (i.e. mortality in transplant cohort), Schoenfeld residual plots for each variable were individually inspected to further assess nonproportionality per Therneau and Grambsch^22^ (Supplementary Figs. 2–4).

Adjusted hazard ratios (AHRs) for death-censored graft failure and graft failure-censored mortality were assessed using cause specific hazard model^23^. Subgroup analyses were completed to assess the effect of key constituents of each GKD group (e.g. polycystic kidney disease for majority monogenic and reflux nephropathy for minority monogenic GKD) on the overall hazard ratios.

Only complete cases were included in the analyses. For all analyses, a significance level of 0.05 was used. Statistical analyses were performed using SPSS software (IBM Corp. Released 2021. IBM SPSS Statistics for Windows, Version 28.0. Armonk, NY: IBM Corp). Study was reported per the Strengthening the Reporting of Observational Studies in Epidemiology (STROBE) Statement: guidelines for reporting observational studies^24^.

### Supplementary Information

Supplementary Information.

## Data Availability

Data stored in ANZDATA is collected and stored on behalf of all Australian and New Zealand kidney units. Due to the ANZDATA data usage agreement, the data cannot be released publicly. However, applications to access this data can be made to the ANZDATA registry available at https://www.anzdata.org.au/anzdata/services/data-policies/.
