# Supplementary material for: People with genetic kidney diseases on kidney replacement therapy have different clinical outcomes compared to people with other kidney diseases
Source: Sci Rep. 2024 Mar 21;14:6746. doi: 10.1038/s41598-024-57273-x (PMC10954746; doi:10.1038/s41598-024-57273-x)
Supplement: Supplementary file 1 — Supplementary Information. [file 41598_2024_57273_MOESM1_ESM.docx]

**People with genetic kidney diseases on kidney replacement therapy have different clinical outcomes compared to people with other kidney diseases**

Helen Y Han, Venkat Vangaveti, Matthew Jose, Monica Suet Ying Ng, Andrew John Mallett

**Supplementary materials**

**Supplementary Table S1: Breakdown of sample size for kidney disease classification**

| **Primary renal disease** | **Dialysis**  **N= (%)** | **Transplant**  **N= (%)** |
| --- | --- | --- |
| **Majority monogenic PRD** |  |  |
| Familial GN [100 +191] | 123 (5.3) | 348 (10.0) |
| Polycystic kidney disease [400] | 2136 (91.9) | 2866 (82.7) |
| Medullary cystic disease [401] | 49 (2.1) | 170 (4.9) |
| Infantile/juvenile polycystic kidney disease [402] | 13 (0.6) | 47 (1.4) |
| Cystinosis [013] | 4 (0.2) | 35 (1.0) |
| Mesangiocapillary GN (Double Contour) [121] | 0 (0) | 0 (0) |
| **Minority monogenic PRD** |  |  |
| Primary focal sclerosis GN/focal glomerular sclerosis [111] | 615 (29.3) | 408 (14.3) |
| Reflux nephropathy [500] | 872 (41.5) | 1703 (59.6) |
| Haemolytic uraemic syndrome [006] | 150 (7.1) | 124 (4.3) |
| Congenital renal hypoplasia + dysplasia [009] | 114 (5.4) | 344 (12.0) |
| Oxalosis [012] | 31 (1.5) | 22 (0.8) |
| **Other PRD** |  |  |
| Advanced GN (unclassified-End Stage) [170] | 339 (0.6) | 284 (1.8) |
| Amyloid disease [005] | 576 (1.1) | 43 (0.3) |
| Analgesic nephropathy [200] | 1839 (3.4) | 218 (1.4) |
| Atheroembolic disease (cholesterol emboli) [303] | 331 (0.6) | 6 (0.01) |
| Balkan nephropathy [014] | 6 (0.01) | 4 (0.01) |
| Bilateral renal artery stenosis [304] | 495 (0.9) | 26 (0.2) |
| Bladder neck obstruction (incl. prostate) [037] | 127 (0.2) | 16 (0.1) |
| Cadmium toxicity [003] | 4 (0.01) | 3 (0.02) |
| Calcineurin inhibitor toxicity [042] | 185 (0.3) | 53 (0.3) |
| Calculi [700] | 400 (0.7) | 84 (0.5) |
| Cortical necrosis [007] | 189 (0.3) | 94 (0.6) |
| Diabetes type I (Insulin dependent) [801] | 1961 (3.6) | 1398 (9.0) |
| Diabetes type II (non-insulin requiring) [802] | 11656 (21.3) | 1056 (6.8) |
| Diabetes type II (Insulin requiring) [803] | 10008 (18.3) | 871 (5.6) |
| Extra and intra-capillary GN (rapidly progressive) [140] | 266 (0.5) | 168 (1.1) |
| Focal sclerosing GN (including hyalinosis) [110] | 452 (0.8) | 323 (2.1) |
| Focal and segmental proliferative GN [160] | 977 (1.8) | 885 (5.7) |
| GN other (Specify) [190] | 298 (0.5) | 131 (0.8) |
| GN with Systemic Disease (Specify) [180] | 87 (0.2) | 35 (0.2) |
| Goodpastures with Linear IgG and Lung Haemorrhage [181] | 164 (0.3) | 158 (1.0) |
| Gout [701] | 69 (0.1) | 24 (0.20) |
| Henoch-Schonlein purpura [184] | 39 (0.1) | 77 (0.5) |
| Interstitial nephritis [008] | 663 (1.2) | 286 (1.8) |
| Lead nephropathy [002] | 66 (0.1) | 16 (0.1) |
| Light chain nephropathy (not malignant) [018] | 77 (0.1) | 13 (0.1) |
| Lithium toxicity [019] | 346 (0.6) | 91 (0.6) |
| Loss of single kidney (trauma-surgery) [010] | 98 (0.2) | 34 (0.2) |
| Megaureter [011] | 9 (0.02) | 7 (0.05) |
| Membranous GN [130] | 598 (1.1) | 392 (2.5) |
| Mesangial proliferative (IgA+) [151] | 148 (0.3) | 169 (1.1) |
| Mesangial proliferative (IgA-) [152] | 1692 (3.1) | 3029 (19.5) |
| Mesangial proliferative (no IF studies) [153] | 81 (0.1) | 91 (0.60 |
| Mesangiocapillary GN (Dense deposit disease) [122] | 62 (0.1) | 57 (0.4) |
| Microscopic polyarteritis [186] | 282 (0.5) | 105 (0.7) |
| Neuropathic bladder [034] | 51 (0.1) | 50 (0.3) |
| Non-obstructed dilated bladder (Megacystitis-megaureter) [035] | 0 (0) | 6 (0.05) |
| Not specified | 185 (0.3) | 45 (0.30) |
| Obstructed megaureter [033] | 12 (0.02) | 6 (0.03) |
| Obstructive nephropathy [041] | 541 (1.0) | 99 (0.6) |
| Other (Specify) [000] | 1519 (2.8) | 484 (3.1) |
| Other lower urinary tract abnormalities [039] | 43 (0.1) | 64 (0.4) |
| Paraproteinaemia (including multiple myeloma) [017] | 1034 (1.9) | 9 (0.1) |
| Pelvic-ureteric junction obstruction [032] | 34 (0.1) | 23 (0.1) |
| Post-partum nephropathy [020] | 10 (0.02) | 16 (0.1) |
| Posterior urethral valves [031] | 14 (0.02) | 151 (1.0) |
| Presumed GN (no biopsy) | 2649 (4.8) | 1322 (8.5) |
| Proliferative GN with linear IgG and no lung haemorrhage [182] | 86 (0.2) | 57 (0.4) |
| Pyelonephritis [600] | 63 (0.1) | 14 (0.1) |
| Renal cell carcinoma [015] | 397 (0.7) | 53 (0.3) |
| Renal vascular disease (malignant hypertension) [300] | 41 (0.1) | 9 (0.1) |
| Renal vascular disease – hypertension (nephrosclerosis) [302] | 663 (1.2) | 205 (1.3) |
| Renal vascular disease – type unspecified [301] | 5231 (9.5) | 807 (5.2) |
| Renal tuberculosis [004] | 2266 (4.1) | 143 (0.9) |
| S.L.E [183] | 432 (0.8) | 424 (2.7) |
| Sarcoidosis [021] | 26 (0.03) | 14 (0.1) |
| Scleroderma [187] | 188 (0.3) | 26 (0.2) |
| Secondary focal sclerosing GN [112] | 128 (0.2) | 67 (0.4) |
| Spina bifida or myelomeningocoele [036] | 37 (0.1) | 47 (0.3) |
| Transitional cell carcinoma urinary tract [016] | 150 (0.3) | 11 (0.1) |
| Uncertain diagnosis [001] | 3615 (6.6) | 870 (5.6) |
| Ureteric obstructive nephropathy [040] | 200 (0.4) | 38 (0.2) |
| Wegeners granulomatosis [185] | 320 (0.6) | 118 (0.8) |
| **Abbreviations**: FSGS=focal segmental glomerulosclerosis, GN=glomerulonephritis, IgA=immunoglobulin A, IgG=immunoglobulin B, MM=multiple myeloma, PRD=primary renal disease 44 | | |

**Supplementary Table S2: Unadjusted and adjusted hazard ratios and 95% CI for covariates in 10-year mortality model for dialysis cohort**

| **Effect** | **Unadjusted** | | **Adjusted** | |
| --- | --- | --- | --- | --- |
|  | **HR** | **95% CI** | **AHR** | **95% CI** |
| **Age** | 1.018*** | 1.017-1.019 | 1.01*** | 1.016-1.019 |
| **Gender** |  | |  | |
| Male | Ref | | Ref | |
| Female | 0.94*** | 0.92-0.96 | 1.01 | 0.98-1.04 |
| **Ethnicity** |  | |  | |
| White | Ref | |  | |
| Non-white | 0.71*** | 0.70-0.73 | 0.84*** | 0.81-0.86 |
| **Smoking status** |  | |  | |
| Never | Ref | |  | |
| Former | 1.16*** | 1.13-1.19 | 1.05*** | 1.02-1.08 |
| Current | 1.07*** | 1.03-1.10 | 1.17*** | 1.12-1.22 |
| **BMI (**kg/m^2^) |  | |  | |
| <18.5 | Ref | |  | |
| 18.5-24.9 | 0.91** | 0.85-0.97 | 0.82*** | 0.76-0.88 |
| 25-29.9 | 0.85*** | 0.80-0.91 | 0.75*** | 0.70-0.81 |
| >30 | 0.71*** | 0.67-0.76 | 0.71*** | 0.66-0.76 |
| **Comorbidities** |  | |  | |
| Lung disease | 1.31*** | 1.27-1.35 | 1.18*** | 1.14-1.22 |
| Coronary artery disease | 1.50*** | 1.47-1.54 | 1.16*** | 1.13-1.19 |
| Peripheral vascular disease | 1.56*** | 1.52-1.60 | 1.20*** | 1.16-1.24 |
| Cerebrovascular disease | 1.42*** | 1.37-1.46 | 1.15*** | 1.11-1.19 |
| Diabetes mellitus | 1.10*** | 1.08-1.13 | 1.26*** | 1.23-1.30 |
| **Dialysis modality** |  | |  | |
| Haemodialysis | Ref | | Ref | |
| Peritoneal dialysis | 1.15*** | 1.13-1.18 | 1.12*** | 1.09-1.15 |
| **Dialysis vintage** |  | |  | |
| 1989-1998 | Ref | | Ref | |
| 1999-2008 | 0.82*** | 0.79-0.84 | 0.80*** | 0.77-0.83 |
| 2009-2018 | 0.57*** | 0.55-0.58 | 0.57*** | 0.55-0.60 |
| 2019-2021 | 0.05*** | 0.048-0.06 | 0.06*** | 0.05-0.07 |
| **Abbreviations**: AHR = adjusted hazard ratio, BMI = body mass index, HR = hazard ratio; NA=not applicable; Significance level: *<0.05, **<0.01, ***<0.001 | | | | |

**Supplementary Table S3: Unadjusted and adjusted hazard ratios and 95% CI for covariates in 10-year mortality model for transplant cohort**

| **Effect** | **Unadjusted** | | **Adjusted** | |
| --- | --- | --- | --- | --- |
|  | **HR** | **95% CI** | **AHR** | **95% CI** |
| **Recipient age** | 1.041*** | 1.038-1.044 | 1.04*** | 1.03-1.04 |
| **Recipient gender** |  | |  | |
| Male | Ref | | Ref | |
| Female | 0.86*** | 0.80-0.93 | 1.00 | 0.90-1.10 |
| **Recipient Ethnicity** |  | |  | |
| White | Ref | | Ref | |
| Non-white | 0.85*** | 0.78-0.93 | 0.81*** | 0.72-0.91 |
| **Recipient Smoking status** |  | |  | |
| Never | Ref | | Ref | |
| Former | 1.55*** | 1.42-1.69 | 1.13** | 1.03-1.25 |
| Current | 1.68*** | 1.49-1.89 | 1.57*** | 1.37-1.80 |
| **Recipient BMI (**kg/m^2^) |  | |  | |
| <18.5 | Ref | | Ref | |
| 18.5-24.9 | 1.45*** | 1.18-1.78 | 0.63*** | 0.50-0.79 |
| 25-29.9 | 1.72*** | 1.40-2.12 | 0.61*** | 0.48-0.77 |
| >30 | 2.00*** | 1.62-2.46 | 0.72** | 0.57-0.91 |
| **Recipient comorbidities** |  | |  | |
| Lung disease | 1.65*** | 1.39-1.96 | 1.19 | 0.98-1.44 |
| Coronary artery disease | 2.65*** | 2.39-2.94 | 1.40*** | 1.23-1.59 |
| Peripheral vascular disease | 2.76*** | 2.41-3.16 | 1.42*** | 1.20-1.67 |
| Cerebrovascular disease | 2.39*** | 2.02-2.82 | 1.42*** | 1.16-1.73 |
| Diabetes mellitus | 2.29*** | 2.11-2.49 | 1.86*** | 1.67-2.08 |
| **First KRT modality** |  | |  | |
| Haemodialysis | Ref | | Ref | |
| Peritoneal dialysis | 0.93 | 0.85-1.01 | 0.99 | 0.90-1.10 |
| Pre-emptive transplant | 0.54*** | 0.46-0.63 | 0.91 | 0.76-1.10 |
| **Dialysis vintage (years)** |  |  |  |  |
| 1989-1998 | Ref | | Ref | |
| 1999-2008 | 0.66*** | 0.61-0.73 | 1.21* | 1.02-1.43 |
| 2009-2018 | 0.52*** | 0.48-0.58 | 1.19 | 0.95-1.50 |
| 2019-2021 | 0.058** | 0.008-0.416 | 0.47 | 0.06-3.54 |
| **Donor source** |  | |  | |
| Deceased | Ref | | Ref | |
| Live donor | 0.59*** | 0.54-0.65 | 1.09 | 0.93-1.27 |
| **Donor age** | 1.007*** | 1.004-1.009 | 1.009*** | 1.006-1.012 |
| **Cold ischaemia time (hours)** | 1.043*** | 1.038-1.049 | 1.02*** | 1.01-1.03 |
| **HLA mismatch** |  |  |  |  |
| 0 Mismatch | Ref | |  | |
| 1-3 Mismatch | 1.33** | 1.09-1.62 | 1.23 | 0.98-1.55 |
| 4-6 Mismatch | 1.34** | 1.100-1.64 | 1.31* | 1.04-1.64 |
| **Transplant era** |  | |  | |
| 1989-1998 | Ref | | Ref | |
| 1999-2008 | 0.57*** | 0.52-0.63 | 0.51*** | 0.43-0.61 |
| 2009-2018 | 0.47*** | 0.43-0.52 | 0.30*** | 0.23-0.37 |
| 2019-2021 | 0.20*** | 0.14-0.29 | 0.12*** | 0.08-0.19 |
| **Abbreviations**: AHR = adjusted hazard ratio, BMI = body mass index, KRT = kidney replacement therapy, HR = hazard ratio; NA=not applicable; Significance level: *<0.05, **<0.01, ***<0.001 | | | | |

**Supplementary Table S4: Competing risk analysis – adjusted Hazard Ratios and 95% confidence intervals for graft failure-censored mortality risk and death-censored graft failure.**

| **Effect** | **Mortality** | | **Graft failure** | |
| --- | --- | --- | --- | --- |
|  | **AHR** | **95% CI** | **AHR** | **95% CI** |
| **Disease status** |  | |  | |
| Majority monogenic | 0.79* | 0.63-0.98 | 0.77*** | 0.68-0.87 |
| Minority monogenic | 1.08 | 0.87-1.34 | 0.96 | 0.84-1.11 |
| Other | Ref | | Ref | |
| **Recipient age (10 years)** | 1.00 | 0.99-1.00 | 1.03*** | 1.03-1.04 |
| **Female gender** | 1.06 | 0.91-1.22 | 0.96 | 0.88-1.05 |
| **Recipient ethnicity** |  | |  | |
| White | Ref | | Ref | |
| Non-white | 1.00 | 0.85-1.17 | 0.91 | 0.82-1.00 |
| **Smoking status** |  | |  | |
| Never | Ref | | Ref | |
| Former | 1.24** | 1.06-1.44 | 1.12** | 1.03-1.23 |
| Current | 1.48*** | 1.19-1.83 | 1.86*** | 1.66-2.09 |
| **BMI** |  | |  | |
| <18.5 | Ref | | Ref | |
| 18.5-24.9 | 0.88 | 0.64-1.20 | 0.611*** | 0.50-0.73 |
| 25-29.9 | 0.94 | 0.68-1.31 | 0.64*** | 0.53-0.78 |
| >30 | 1.01 | 0.71-1.42 | 0.81* | 0.66-0.99 |
| **Lung disease** | 1.01** | 0.72-1.41 | 1.12 | 0.93-1.34 |
| **Coronary artery disease** | 1.33** | 1.07-1.66 | 1.28*** | 1.14-1.44 |
| **Peripheral vascular disease** | 1.17 | 0.87-1.56 | 1.35*** | 1.15-1.59 |
| **Cerebrovascular disease** | 1.72*** | 1.26-2.36 | 1.47*** | 1.22-1.77 |
| **Diabetes mellitus** | 1.79*** | 1.50-2.12 | 1.7*** | 1.59-1.94 |
| **Donor source** |  | |  | |
| Deceased | Ref | | Ref | |
| Live donor | 0.94 | 0.74-1.18 | 0.86* | 0.75-0.98 |
| **Dialysis vintage (years)** |  | |  | |
| 1989-1998 | Ref | | Ref | |
| 1999-2008 | 1.02 | 0.76-1.37 | 0.64*** | 0.56-0.72 |
| 2009-2018 | 3.6*** | 2.4-5.4 | 0.41*** | 0.34-0.49 |
| 2019-2021 | 14.8*** | 6.06-36.30 | 022 | 0.02-1.74 |
| **HLA mismatch** |  | |  | |
| 0 Mismatch | Ref | | Ref | |
| 1-3 Mismatch | 1.28 | 1.01-1.02 | 1.29** | 1.05-1.58 |
| 4-6 Mismatch | 1.58** | 1.08-1.04 | 1.49*** | 1.21-1.82 |
| **Donor age (10 years)** | 1.019*** | 1.015-1.024 | 1.010*** | 1.008-1.013 |
| **Cold ischaemia time (hours)** | 1.02*** | 1.01-1.04 | 1.014*** | 1.005-1.023 |
| **Transplant era** |  | |  | |
| 1989-1998 | Ref | | Ref | |
| 1999-2008 | 0.45*** | 0.34-0.60 | 0.91 | 0.80-1.04 |
| 2009-2018 | 0.23*** | 0.15-0.35 | 0.78** | 0.65-0.94 |
| 2018-2021 | 0.23*** | 0.13-.0.40 | 0.42** | 0.22-0.80 |
| **Abbreviations**: AHR = adjusted hazard ratio, ATSI = Aboriginal and Torres Strait Islander, BMI = body mass index, CI = confidence intervals, KRT = kidney replacement therapy; Significance level: *<0.05, **<0.01, ***<0.001 | | | | |

**Supplementary Table S5: Unadjusted and adjusted hazard ratios and 95% CI for covariates in 10-year graft failure model**

| **Effect** | **Unadjusted** | | **Adjusted** | |
| --- | --- | --- | --- | --- |
|  | **HR** | **95% CI** | **AHR** | **95% CI** |
| **Recipient age** | 0.980*** | 0.978-0.982 | 0.97*** | 0.96-0.97 |
| **Recipient Gender** |  | |  | |
| Male | Ref | | Ref | |
| Female | 1.01 | 0.94-1.10 | 1.07 | 0.98-1.17 |
| **Recipient Ethnicity** |  | |  | |
| White | Ref | |  | |
| Non-white | 1.42*** | 1.31-1.53 | 1.20*** | 1.08-1.32 |
| **Smoking status** |  | |  | |
| Never | Ref | |  | |
| Former | 1.01 | 0.92-1.10 | 1.16** | 1.05-1.28 |
| Current | 1.74*** | 1.55-1.95 | 1.78*** | 1.57-2.01 |
| **BMI (**kg/m^2^) |  | |  | |
| <18.5 | Ref | |  | |
| 18.5-24.9 | 0.71*** | 0.62-0.82 | 0.99 | 0.84-1.16 |
| 25-29.9 | 0.69*** | 0.59-0.80 | 1.12 | 0.94-1.34 |
| >30 | 0.78** | 0.67-0.92 | 1.27** | 1.05-1.53 |
| **Recipient comorbidities** |  | |  | |
| Lung disease | 0.98 | 0.78-1.22 | 1.03 | 0.81-1.31 |
| Coronary artery disease | 0.99 | 0.84-1.15 | 1.14 | 0.95-1.37 |
| Peripheral vascular disease | 1.00 | 0.80-1.24 | 0.98 | 0.76-1.26 |
| Cerebrovascular disease | 0.97 | 0.75-1.26 | 1.23 | 0.92-1.63 |
| Diabetes mellitus | 1.16** | 1.05-1.29 | 1.25*** | 1.10-1.43 |
| **First KRT modality** |  | |  | |
| Haemodialysis | Ref | |  | |
| Peritoneal dialysis | 0.96 | 0.88-1.04 | 0.94 | 0.86-1.04 |
| Pre-emptive transplant | 0.63*** | 0.55-0.73 | 0.81* | 0.69-0.96 |
| **Dialysis vintage (years)** |  |  |  |  |
| 1989-1998 | Ref | | Ref | |
| 1999-2008 | 0.74*** | 0.68-0.81 | 0.57*** | 0.50-0.65 |
| 2009-2018 | 0.57*** | 0.51-0.63 | 0.38*** | 0.31-0.47 |
| 2019-2021 | 0.11*** | 0.01-0.81 | 0.17 | 0.02-1.38 |
| **Donor source** |  | |  | |
| Deceased | Ref | | Ref | |
| Live donor | 0.78*** | 0.72-0.84 | 0.78*** | 0.66-0.91 |
| **Donor age** | 1.011*** | 1.008-1.013 | 1.020*** | 1.017-1.023 |
| **Cold ischaemia time (hours)** | 1.018*** | 1.012-1.023 | 1.00 | 0.99-1.01 |
| **HLA mismatch** |  |  |  |  |
| 0 Mismatch | Ref | |  | |
| 1-3 Mismatch | 1.52*** | 1.25-1.85 | 1.20 | 0.96-1.49 |
| 4-6 Mismatch | 1.63*** | 1.34-1.98 | 1.48*** | 1.19-1.85 |
| **Transplant era** |  | |  | |
| 1989-1998 | Ref | | Ref | |
| 1999-2008 | 0.78*** | 0.71-0.85 | 1.33*** | 1.15-1.53 |
| 2009-2018 | 0.62*** | 0.56-0.68 | 1.66*** | 1.37-2.03 |
| 2019-2021 | 0.36*** | 0.22-0.60 | 1.33 | 0.72-2.47 |
| **Abbreviations**: AHR = adjusted hazard ratio, ATSI = Aboriginal and Torres Strait Islander, BMI = body mass index, HR = hazard ratio, KRT = kidney replacement therapy; NA=not applicable  Significance level: *<0.05, **<0.01, ***<0.001 | | | | |

**Supplementary Table S6: Subgroup analysis of GKD and 10-year mortality in the dialysis cohort**

| **Effect** | **Unadjusted** | | **Adjusted** | |
| --- | --- | --- | --- | --- |
|  | **HR** | **95% CI** | **AHR** | **95% CI** |
| **Disease status** |  | |  | |
| Polycystic kidney disease | 0.68*** | 0.64-0.73 | 0.70*** | 0.66-0.76 |
| Other majority monogenic | 0.59*** | 0.48-0.74 | 0.71** | 0.55-0.91 |
| Reflux nephropathy | 0.71*** | 0.65-0.78 | 0.85** | 0.76-0.94 |
| Other minority monogenic | 0.64*** | 0.58-0.70 | 0.87** | 0.78-0.96 |
| Other kidney diseases | Ref | | Ref | |
| **Age** | 1.018*** | 1.017-1.019 | 1.018*** | 1.016-1.019 |
| **Gender** |  | |  | |
| Male | Ref | | Ref | |
| Female | 0.94*** | 0.92-0.96 | 1.01 | 0.98-1.04 |
| **Ethnicity** |  | |  | |
| White | Ref | |  | |
| Non-white | 0.71*** | 0.70-0.73 | 0.84*** | 0.81-0.86 |
| **Smoking status** |  | |  | |
| Never | Ref | |  | |
| Former | 1.16*** | 1.13-1.19 | 1.05*** | 1.02-1.08 |
| Current | 1.07*** | 1.03-1.10 | 1.17*** | 1.12-1.22 |
| **BMI (**kg/m^2^) |  | |  | |
| <18.5 | Ref | |  | |
| 18.5-24.9 | 0.91** | 0.85-0.97 | 0.82*** | 0.76-0.88 |
| 25-29.9 | 0.85*** | 0.80-0.91 | 0.75*** | 0.70-0.81 |
| >30 | 0.71*** | 0.67-0.76 | 0.71*** | 0.66-0.76 |
| **Comorbidities** |  | |  | |
| Lung disease | 1.31*** | 1.27-1.35 | 1.18*** | 1.14-1.22 |
| Coronary artery disease | 1.50*** | 1.47-1.54 | 1.16*** | 1.13-1.19 |
| Peripheral vascular disease | 1.56*** | 1.52-1.60 | 1.20*** | 1.16-1.24 |
| Cerebrovascular disease | 1.42*** | 1.37-1.46 | 1.15*** | 1.11-1.19 |
| Diabetes mellitus | 1.10*** | 1.08-1.13 | 1.26*** | 1.23-1.30 |
| **First KRT modality** |  | |  | |
| Haemodialysis | Ref | |  | |
| Peritoneal dialysis | 1.15*** | 1.13-1.18 | 1.12*** | 1.09-1.15 |
| **Dialysis vintage** |  | |  | |
| 1989-1998 | Ref | | Ref | |
| 1999-2008 | 0.82*** | 0.79-0.84 | 0.80*** | 0.77-0.83 |
| 2009-2018 | 0.57*** | 0.55-0.58 | 0.57*** | 0.55-0.60 |
| 2019-2021 | 0.05*** | 0.048-0.06 | 0.36*** | 0.05-0.07 |
| **Abbreviations**: AHR = adjusted hazard ratio, ATSI = Aboriginal and Torres Strait Islander, BMI = body mass index, HR = hazard ratio, KRT = kidney replacement therapy, HR = hazard ratio; NA=not applicable; Significance level: *<0.05, **<0.01, ***<0.001 | | | | |

**Supplementary Table S7: Subgroup analysis of GKD and 10-year mortality in the transplant cohort**

| **Effect** | **Unadjusted** | | **Adjusted** | |
| --- | --- | --- | --- | --- |
|  | **HR** | **95% CI** | **AHR** | **95% CI** |
| **Disease status** |  | |  | |
| Polycystic kidney disease | 0.77*** | 0.69-0.87 | 0.82** | 0.72-0.95 |
| Other majority monogenic | 0.37*** | 0.26-0.52 | 0.75 | 0.51-1.10 |
| Reflux nephropathy | 0.43*** | 0.36-0.52 | 0.73** | 0.59-0.90 |
| Other minority monogenic | 0.50*** | 0.40-0.63 | 0.95 | 0.74-1.23 |
| Other kidney diseases | Ref | | Ref | |
| **Recipient age** | 1.041*** | 1.038-1.044 | 1.043*** | 1.038-1.047 |
| **Recipient gender** |  | |  | |
| Male | Ref | | Ref | |
| Female | 0.86*** | 0.80-0.93 | 1.01 | 0.91-1.10 |
| **Recipient Ethnicity** |  | |  | |
| White | Ref | | Ref | |
| Non-white | 0.85*** | 0.78-0.93 | 0.81*** | 0.72-0.90 |
| **Recipient Smoking status** |  | |  | |
| Never | Ref | | Ref | |
| Former | 1.55*** | 1.42-1.69 | 1.13** | 1.03-1.25 |
| Current | 1.68*** | 1.49-1.89 | 1.58*** | 1.37-1.81 |
| **Recipient BMI (**kg/m^2^) |  | |  | |
| <18.5 | Ref | | Ref | |
| 18.5-24.9 | 1.45*** | 1.18-1.78 | 0.63*** | 0.50-0.79 |
| 25-29.9 | 1.72*** | 1.40-2.12 | 0.61*** | 0.48-0.77 |
| >30 | 2.00*** | 1.62-2.46 | 0.72*** | 0.57-0.91 |
| **Recipient comorbidities** |  | |  | |
| Lung disease | 1.65*** | 1.39-1.96 | 1.19 | 0.98-1.44 |
| Coronary artery disease | 2.65*** | 2.39-2.94 | 1.40*** | 1.23-1.59 |
| Peripheral vascular disease | 2.76*** | 2.41-3.16 | 1.42*** | 1.20-1.67 |
| Cerebrovascular disease | 2.39*** | 2.02-2.82 | 1.41*** | 1.16-1.73 |
| Diabetes mellitus | 2.29*** | 2.11-2.49 | 1.86*** | 1.67-2.08 |
| **First KRT modality** |  | |  | |
| Haemodialysis | Ref | | Ref | |
| Peritoneal dialysis | 0.93 | 0.85-1.01 | 0.99 | 0.90-1.10 |
| Pre-emptive transplant | 0.54*** | 0.46-0.63 | 0.92 | 0.76-1.11 |
| **Dialysis vintage (years)** |  |  |  |  |
| 1989-1998 | Ref | | Ref | |
| 1999-2008 | 0.74*** | 0.68-0.81 | 1.21** | 1.02-1.44 |
| 2009-2018 | 0.57*** | 0.51-0.63 | 1.19 | 0.95-1.50 |
| 2019-2021 | 0.11*** | 0.01-0.81 | 0.47 | 0.06-3.54 |
| **Donor source** |  | |  | |
| Deceased | Ref | | Ref | |
| Live donor | 0.59*** | 0.54-0.65 | 1.09 | 0.93-1.27 |
| **Donor age** | 1.007*** | 1.004-1.009 | 1.009*** | 1.006-1.012 |
| **Cold ischaemia time (hours)** | 1.043*** | 1.038-1.049 | 1.02*** | 1.01-1.03 |
| **HLA mismatch** |  |  |  |  |
| 0 Mismatch | Ref | | Ref | |
| 1-3 Mismatch | 1.33** | 1.09-1.62 | 1.23 | 0.98-1.55 |
| 4-6 Mismatch | 1.34** | 1.100-1.64 | 1.31** | 1.04-1.64 |
| **Transplant era** |  | |  | |
| 1989-1998 | Ref | | Ref | |
| 1999-2008 | 0.57*** | 0.52-0.63 | 0.51*** | 0.42-0.60 |
| 2009-2018 | 0.47*** | 0.43-0.52 | 0.29*** | 0.23-0.37 |
| 2019-2021 | 0.20*** | 0.14-0.29 | 0.12*** | 0.07-0.19 |
| **Abbreviations**: AHR = adjusted hazard ratio, ATSI = Aboriginal and Torres Strait Islander, BMI = body mass index, HR = hazard ratio, KRT = kidney replacement therapy; NA=not applicable; Significance level: *<0.05, **<0.01, ***<0.001 | | | | |

**Supplementary Table S8: Subgroup analysis of GKD and 10-year graft failure in the transplant cohort**

| **Effect** | **Unadjusted** | | **Adjusted** | |
| --- | --- | --- | --- | --- |
|  | **HR** | **95% CI** | **AHR** | **95% CI** |
| **Disease status** |  | |  | |
| Polycystic kidney disease | 0.43*** | 0.36-0.50 | 0.58*** | 0.48-0.69 |
| Other majority monogenic | 1.10 | 0.90-1.35 | 0.97 | 0.78-1.22 |
| Reflux nephropathy | 1.02 | 0.90-1.16 | 0.99 | 0.86-1.15 |
| Other minority monogenic | 1.41 | 1.21-1.63 | 1.34*** | 1.13-1.58 |
| Other kidney diseases | Ref | | Ref | |
| **Recipient age (10 years)** | 0.980*** | 0.978-0.982 | 0.974*** | 0.971-0.977 |
| **Recipient Gender** |  | |  | |
| Male | Ref | | Ref | |
| Female | 1.01 | 0.94-1.10 | 1.08 | 0.98-1.18 |
| **Recipient Ethnicity** |  | |  | |
| White | Ref | |  | |
| Non-white | 1.42*** | 1.31-1.53 | 1.19*** | 1.08-1.31 |
| **Smoking status** |  | |  | |
| Never | Ref | |  | |
| Former | 1.01 | 0.92-1.10 | 1.16** | 1.05-1.29 |
| Current | 1.74*** | 1.55-1.95 | 1.78*** | 1.57-2.02 |
| **BMI (**kg/m^2^) |  | |  | |
| <18.5 | Ref | |  | |
| 18.5-24.9 | 0.71*** | 0.62-0.82 | 1.02 | 0.87-1.20 |
| 25-29.9 | 0.69*** | 0.59-0.80 | 1.16 | 0.97-1.39 |
| >30 | 0.78** | 0.67-0.92 | 1.31** | 1.08-1.58 |
| **Recipient comorbidities** |  | |  | |
| Lung disease | 0.98 | 0.78-1.22 | 1.02 | 0.80-1.30 |
| Coronary artery disease | 0.99 | 0.84-1.15 | 1.13 | 0.94-1.36 |
| Peripheral vascular disease | 1.00 | 0.80-1.24 | 0.98 | 0.76-1.26 |
| Cerebrovascular disease | 0.97 | 0.75-1.26 | 1.23 | 0.93-1.64 |
| Diabetes mellitus | 1.16** | 1.05-1.29 | 1.24*** | 1.09-1.41 |
| **First KRT modality** |  | |  | |
| Haemodialysis | Ref | |  | |
| Peritoneal dialysis | 0.96 | 0.88-1.04 | 0.94 | 0.85-1.04 |
| Pre-emptive transplant | 0.63*** | 0.55-0.73 | 0.82* | 0.69-0.97 |
| **Dialysis vintage (years)** |  |  |  |  |
| 1989-1998 | Ref | | Ref | |
| 1999-2008 | 0.74*** | 0.68-0.81 | 0.57*** | 0.50-0.65 |
| 2009-2018 | 0.57*** | 0.51-0.63 | 0.38*** | 0.31-0.47 |
| 2019-2021 | 0.11*** | 0.01-0.81 | 0.17 | 0.02-1.39 |
| **Donor source** |  | |  | |
| Deceased | Ref | | Ref | |
| Live donor | 0.78*** | 0.72-0.84 | 0.78** | 0.67-0.91 |
| **Donor age (10 years)** | 1.011*** | 1.008-1.013 | 1.020*** | 1.017-1.023 |
| **Cold ischaemia time (hours)** | 1.018*** | 1.012-1.023 | 1.00 | 0.99-1.01 |
| **HLA mismatch** |  |  |  |  |
| 0 Mismatch | Ref | |  | |
| 1-3 Mismatch | 1.52*** | 1.25-1.85 | 1.20 | 0.97-1.49 |
| 4-6 Mismatch | 1.63*** | 1.34-1.98 | 1.48*** | 1.19-1.85 |
| **Transplant era** |  | |  | |
| 1989-1998 | Ref | | Ref | |
| 1999-2008 | 0.78*** | 0.71-0.85 | 1.32*** | 1.14-1.52 |
| 2009-2018 | 0.62*** | 0.56-0.68 | 1.65*** | 1.35-2.01 |
| 2019-2021 | 0.36*** | 0.22-0.60 | 1.31 | 0.70-2.43 |
| **Abbreviations**: AHR = adjusted hazard ratio, ATSI = Aboriginal and Torres Strait Islander, BMI = body mass index, HR = hazard ratio, KRT = kidney replacement therapy; NA=not applicable; Significance level: *<0.05, **<0.01, ***<0.001 | | | | |

**Supplementary Figure 1: Log-log survival curves for (A) Mortality in Dialysis cohort, (B) Mortality in Transplant cohort and (C) Graft failure analyses**

**Supplementary Figure 2: Schoenfeld residual plots for each variable for mortality in dialysis cohort (A) Majority monogenic, (B) minority monogenic, (C) Age at RRT entry, (D) Female gender, (E) Current smoker, (F) Former smoker, (G) Non-white ethnicity, (H) BMI 18.5-24.9, (I) BMI 25-29.9, (J) BMI >30, (K) Chronic lung disease, (L) Coronary artery disease, (M) Peripheral vascular disease, (N) Cerebrovascular disease, (O) Diabetes, (P) Peritoneal dialysis, (Q) Dialysis vintage 1999-2008, (R) Dialysis vintage 2009-2018, (S) Dialysis vintage 2019-2021**

**Supplementary Figure 3: Schoenfeld residual plots for each variable for mortality in transplant cohort (A) Majority monogenic, (B) Minority monogenic, (C) Age at transplant, (D) Female gender, (E) Non-white, (F) Former smoker, (G) Current smoker, (H) BMI 18.5-24.9, (I) BMI 25-29.9, (J) BMI >30, (K) Chronic lung disease, (L) Coronary artery disease, (M) Peripheral vascular disease, (N) Cerebrovascular disease, (O) Diabetes, (P) Peritoneal dialysis, (Q) Pre-emptive transplant, (R) Dialysis vintage 1999-2008, (S) Dialysis vintage 2009-2018, (T) Dialysis vintage 2019-2021, (U) Live donor, (V) Donor age, (W) Cold ischaemia time, (X) HLA 1-3 mismatch, (Y) HLA 4-6 mismatch, (Z) Transplant era 1999-2008, (AA) Transplant era 2009-2018, (AB) Transplant era 2019-2021**

**Supplementary Figure 4: Schoenfeld residual plots for each variable for graft failure (A) Majority monogenic, (B) Minority monogenic, (C) Age at transplant, (D) Female gender, (E) Non-white, (F) Former smoker, (G) Current smoker, (H) BMI 18.5-24.9, (I) BMI 25-29.9, (J) BMI >30, (K) Chronic lung disease, (L) Coronary artery disease, (M) Peripheral vascular disease, (N) Cerebrovascular disease, (O) Diabetes, (P) Peritoneal dialysis, (Q) Pre-emptive transplant, (R) Dialysis vintage 1999-2008, (S) Dialysis vintage 2009-2018, (T) Dialysis vintage 2019-2021, (U) Live donor, (V) Donor age, (W) Cold ischaemia time, (X) HLA 1-3 mismatch, (Y) HLA 4-6 mismatch, (Z) Transplant era 1999-2008, (AA) Transplant era 2009-2018, (AB) Transplant era 2019-2021**
